# The prevalence of asymptomatic malaria parasitemia and associated factors among adults in Dembia district, northwest Ethiopia, 2017

**DOI:** 10.1186/s13690-018-0323-z

**Published:** 2018-12-20

**Authors:** Mesafint Fekadu, Melaku Kindie Yenit, Ayenew Molla Lakew

**Affiliations:** 1 North Gondar Zone Public Health Emergency Management, Gondar, Ethiopia; 2 Department of Epidemiology and Biostatistics, Institute of Public Health, University of Gondar, Po.Box: 196, Gondar, Ethiopia

**Keywords:** Malaria, Adult, Northwest Ethiopia

## Abstract

**Background:**

Malaria is still a leading cause of morbidity and mortality in many developing countries including Ethiopia. Its prevalence has been declining among Ethiopian adults, especially in Dembia district. However, it is still at the top of diseases list at the district. Hence, the study aimed to determine the prevalence and the factors that contribute to its being the major public health concern despite different preventive and control measures in place.

**Methods:**

A community based cross-sectional study was conducted from October 11 to November 16, 2017. The multistage sampling technique was employed to select 832 study participants. A rapid diagnostic test were used to confirm the disease. Data were entered using Epi info version 7 and was analyzed by Statistical Package for Social Science (SPSS) version 20. The logistic regression model was performed to examine the association of factors with malaria parasite.

**Results:**

Out of the 832 adults included in the study, 6.7% (95% Confidence Interval (CI: 5.2–8.7)) were confirmed to be malaria parasite carriers. The dominant plasmodium species was *Plasmodium falciparum* [46 (82%)]. According to the multivariable logistic regression analysis, male sex (Adjusted Odds Ratio (AOR = 4.5; 95%CI: 2.1–9.5), adult age 15–19 years (AOR = 4.5; 95%CI: 2.1–9.7), travel history (AOR = 5; 95%CI: 2.34–12.25), and stagnant water around home (AOR = 3.7; 95%CI: 1.57–8.87) increased the probability of malaria infectivity, while Insecticidal Treated Nets (ITN) utilization (AOR = 0.2; 95%CI: 0.09–0.31) decreased it.

**Conclusion:**

Malaria is still an important public health challenge among adults in the study area. Male sex, age 15–19 years, travel history, living around stagnant water, and not using ITN increased the probability of infection. Therefore, the District Health office and Health extension workers should work to increase ITN distribution and focus on reducing malaria breading sites through community participation.

## Background

Malaria is one of the most severe public health problems and the leading cause of death in many developing countries. Globally, there were an estimated 214 million new cases of malaria and 438,000 deaths in 2015. Of this, 88% of the cases and 90% of the deaths occurred in the African region [1]. In 2016, there were an estimated 216 million cases and 445,000 deaths attributable to the disease globally. About 85% of the estimated malaria cases (P.vivax) occurred in five countries that includes Ethiopia [2].

Ethiopia is generally considered as a low to moderate malaria transmission intensity country with about 75% of the land and 60% of the people is exposed. Due to the unstable and seasonal transmission of malaria in the country, the protective immunity of the population is generally low causing all age groups to be at risk [1]. Studies conducted in different parts of Ethiopia indicated that the prevalence of malaria in the country ranges from 2.8 to 28.1% [3–9]. In the 2014 annual report the national incidence of malaria was 3540 cases/100000 people. The highest (610,486) case were reported in Amhara region with an annual incidence rate of 3866 per 100,000 [10].

Malaria imposes substantial costs on both individuals and governments. Direct costs (for example, illness, treatment, premature death) have been estimated to be at least USD 12 billion per year globally [11]. Despite substantial cost savings, malaria has placed a heavy economic burden on health systems in Africa. Since 2000, the average annual cost of case management alone has been estimated at nearly USD 300 million [1].

Ethiopia has achieved a remarkable progress in the fight against malaria in the last decade following the launching of the Health Extension Program in 2003 [12]. The major activities for malaria prevention and control focused on expanding vector control, strengthening case detection and treatment, increasing availability and use of Long-Lasting Insecticide-treated Nets (LLIN), and implementing Indoor Residual Spray (IRS) [13]. Despite the declining trend of the prevalence in Ethiopia including Dembia district, malaria is among top short listed diseases [12]. So far, studies conducted to ascertain malaria prevalence and associated factors were institution-based and are not adequately generalizable to communities. Therefore, this study determined the current prevalence of asymptomatic malaria parasitemia and associated factors among adults in the community. The study is valuable for indirectly assessing the effectiveness of interventions aimed at malaria eradication and/or prevention. The study is also expected to help policy makers redesign malaria control and elimination strategies.

## Methods

### Study area and period

A community based cross-sectional study was conducted on the residents of Dembia district, located in North Gondar zone, northwest Ethiopia, from October 11 to November 16, 2017. The total population of the district was about 321,856 of whom 159,962 (49.7%) were male. The district had 45 (40 rural and 5 urban) malarious kebeles. The altitude of the district ranges from 1800 m–2600 m above sea level. The climate of the district was 100% woina-dega, and annual rain fall ranged from 772 mmHg–1260 mmHg. Annual temperature ranged from 18 to 30 degree Celsius. The district had one hospital, 10 health centers, 40 health posts and 20 private clinics at its services.

### Sample size and sampling techniques

All adults (age > =15 years) in Dembia district were taken as a source population, while all adult individuals in randomly selected kebeles were considered as a study participants. Any adult individuals who took anti-malarial drugs 2 weeks before the survey, severely ill and unable to respond were not included.

A sample of 310 adults was estimated using the single population proportion formula and considering a 95% confidence level, 5% margin of error, and 28.1% prevalence of malaria infection [4]. Considering 95% confidence level, 80% power, and 1:1 unexposed to exposed ratio, sample size was also estimated for different significant and pertinent factors for malaria infection, and stagnant water yielded a large sample (378). Then, by adding a 10% non-response rate and using a design effect of 2, 832 participants were estimated for the study.

A multistage sampling technique was used to select representative households. First a list of 45 kebeles was obtained from the District Health Office, at which 9(20%) were selected using the simple random sampling technique and considering as a primary sampling unit. Then the estimated sample was proportionally distributed to the selected kebeles based on their number of households and considering them as a secondary sampling unit. Finally, the lottery method was used to select any single household member for a rapid diagnostic test.

### Data collection procedures

The dependent variable of this study was the malaria test result (Positive, Negative). The independent variables were age, sex, educational status, family size, knowledge about malaria (cause, prevention methods, mode of transmission, signs and symptoms), main source of drinking water**,** travel history, occupation, toilet facilities, outdoor activities, stagnant water**,** ITN possession**,** ITN utilization, and IRS.

Blood samples were drawn and tested for malaria presence using a Rapid Diagnostic Test (RDT). Carestart™ malaria Pf/Pv (HRP2/pLDH) Ag combo RDT was used. The validity of the kit was checked through 2 replicates of carestart™ malaria Pf/Pv (hrp2/pLDH) Ag combo RDT from each box with negative and positive clinical samples confirmed by microscopic examinations. In addition, a semi-structured questionnaire adapted from previous studies was administered to collect data on socio-demographic, economic and environmental factors, prevention methods, and knowledge relating to malaria infection.

Observations have been made on the possession and utilization of ITN and the availability of stagnant water around houses. The data were collected by 8 health extension workers and 2 laboratory technicians, supervised by 2 clinical nurses.

Before the actual data collection, a pretest was administered on 5% of the sample not included in the main study. Both data collectors and supervisors were trained for 3 days on how to approach study subjects and collect data. The supervisors and the principal investigator closely followed the daily completeness and appropriateness of the data collection. The validity of the kit was checked by testing 2 replicates of carestart™ malaria Pf/Pv (hrp2/pLDH) Ag combo RDT before the actual data collection. Knowledge of respondents in this study was measured considering respondents knowledge on the basic concepts of malaria prevention and control strategies such as causes, symptoms of malaria and means of prevention and control mechanisms. Accordingly, the mean score of respondents was used as to classify knowledge as good and poor.

### Data management and analysis

Data were entered into Epi info version 7, exported and analyzed using SPSS version 20. Descriptive statistics, like the median with an interquartile range, frequencies, and percentages were employed. Cross-tabulations were plotted before both bivariate and multivariable logistic regression analyses were performed. Bi-variable and multi-variable logistic regression analyses were performed to examine the associations of covariates with malaria infection. All risk factors with *p*-value < 0.25 at the bi-variable analysis were entered into the multi-variable analysis to control confounding effects. The associations between dependent and independent variables were measured and tested using Odds Ratio (OR) with a 95% CI. The parsimonious model fitness was checked by Hosmer and Lemeshow goodness of fit test and a *P*-value of 0.67 was obtained.

## Results

### Socio-demographic characteristics of participants

A total of 832 individuals participated in this study with a response rate of 100%. Four hundred thirty-seven (52.5%) of the participants were female with about 547 (67.5%) unable to read and write. The median age was 34 (Inter Quartile Range (IQR): 24–45) years. About 64.8% of the households had less than 5 members with the median family size of 4 (IQR: 3–6) (Table 1).

**Table 1:** Socio-demographic and individual characteristics of respondents in Dembia district, North West Ethiopia, 2017

Variables	Frequency	Percentage
Age (in years)		
15–19	75	9.0
> 20	757	91.0
Sex		
Female	437	52.5
Male	395	47.5
Family size		
< 5	539	64.8
> 5	293	35.2
Educational status		
No education	547	65.7
Able to read and write	285	34.3
Sleeping area		
Indoor	802	96.4
Outdoor	30	3.6
Outdoor activities before dawn and dusk		
Yes	356	42.8
No	476	57.2
History of travel to malarious area		
Yes	46	5.5
No	786	94.5
Availability of toilet facility		
Yes	537	64.5
No	295	35.5
Cattle in the house		
Yes	531	63.8
No	301	36.2
Stagnant water around home		
Yes	58	7
No	774	93
ITN possession		
Yes	770	92.5
No	62	7.5
Always sleep under mosquito net		
Yes	687	82.6
No	145	17.4
IRS in the last 6 month		
Yes	277	33.3
No	555	66.7
Source of water		
Protected	555	66.7
Unprotected	277	33.3
Knowledge		
Poor	530	63.7
Good	302	36.3

### Risk factor characteristics of study participants

The majority (92%) of the participants used household ITNs at bed-times. Out of those who had ITNs, 687(89%) always slept under mosquito nets, while only 277 (33%) households sprayed chemicals in the last 6 months. Five hundred thirty (63.7%) of respondents had poor knowledge about malaria signs, symptoms, and its prevention and control methods (Table 1).

### The prevalence of malaria infection

Fifty-six (6.7, 95% CI: 0.052, 0.087) of the participants had RDT confirmed malaria parasites in their blood samples. The dominant plasmodium species was *Plasmodium falciparum* (46(82%)) followed by 5(9%) *Plasmodium vivax*, and (Pf + Pv) 5(9%) mixed infections.

### Factors associated with malaria infection

After adjusting for potential confounding variables, being male sex was 4.5 times more likely to have malaria infections (AOR = 4.5, 95%CI: 2.1–9.5). Individuals in the age group of 15–19 years were 4.5 times more likely to have malaria infections (AOR = 4.5, 95%CI: 2.1–9.7) compared to 20 and above age groups. Those who always slept under mosquito nets decreased the risk of malaria positivity by 80% (AOR = 0.2, 95%CI: 0.09–0.31). Individuals who traveled away from residence had 5 times more chance of having malaria infections (AOR = 5, 95%CI: 2.34–12.25) compared to those who did not. Individuals who lived close to stagnant water (AOR = 3.7, 95%CI: 1.57–8.87) were also significantly associated with malaria infection than those who did not (Table 2).

**Table 2:** Bi-variable and multi-variable regression analysis of covariates related to malaria positivity in Dembia district, North West Ethiopia, 2017

Variables	Malaria test positivity			
	Positive	Negative	COR (95% CI)	AOR (95% CI)
Sex				
Female	10	427	1.00	1.00
Male	46	349	5.6 [2.8–11.3]	4.5 [2.10–9.50]^*^
Age				
15–19	15	60	4.4 [2.3–8.3]	4.5 [2.10–9.70]^*^
> =20	41	716	1.00	1.00
Family size				
< 5	38	501	1.00	
> = 5	18	275	0.9 [0.48–1.54]	
Educational status				
Illiterate	33	514	1.00	
Literate	23	262	0.73 [0.42–1.27]	
Travel history				
Yes	15	31	8.8 [4.40–17.56]	5.3 [2.34–12.25]^*^
No	41	745	1.00	1.00
Sleeping area				
Indoor	48	754	1.00	1.00
Outdoor	8	22	5.7 [2.43–13.50]	1.7 [0.5–5.8]
Outdoor activities				
Yes	28	328	1.4 [0.79–2.35]	
No	28	448	1.00	
Toilet facilities available				
Yes	30	507	1.00	1.00
No	26	269	1.6 [0.95–2.82]	0.68 [0.35–1.29]
Cattle in the house				
Yes	45	578	1.00	
No	11	198	0.7 [0.36–1.41]	
Stagnant water around home				
Yes	13	45	4.911 [2.46–9.79]	3.7 [1.56–8.87]^*^
No	43	731	1.00	1.00
Always sleep under mosquito net				
Yes	26	661	0.15 [0.09–0.26]	0.2 [0.09–0.31]^*^
No	30	115	1.00	1.00
IRS^a^				
Yes	23	254	1.4 [0.82–2.49]	1.3 [0.67–2.6]
No	33	522	1.00	1.00
Knowledge				
Poor	41	519	1.35 [0.73–2.49]	
Good	15	257	1.00	
Source of drinking water				
Protected	33	522	1.00	1.00
Unprotected	23	254	1.4 [0.82–2.49]	1.6 [0.85–3.1]

^a^Integrated residual Spray

^*^variables significant at a *P*-value of less than 0.05

## Discussion

This study showed that malaria is still a public health concern among the adult population of Dembia district. The study revealed that the overall malaria prevalence was 6.7% (95% CI: 5.2–8.7). However, the studies conducted in Dilla town and its surrounding Gedeo zone [8] Hadiya zone [3], Chuchu and Wnago health center [4] and East Shewa zone of the Oromia regional state [9] found a much higher prevalence of malaria. This discrepancy might be due to differences of participants; the other studies were conducted on suspected febrile cases who came for some medical services only. Our finding is higher than that of a study conducted in Jimma town, southwest Ethiopia (5.2%) [7]. This variation might be attributed to different data collection periods; in this study data were collected during the major malaria transmission season. It is also almost three times higher than the 2.8% of a study conducted in Jabi Tehnan district, Amhara Region [5]. The variations could be attributed to different climatic conditions for example, less rain fall and availability of surface water in the study areas serve as mosquito breeding sites.

In this study, *Plasmodium falciparum* which accounted for 82% of the cases was the dominant plasmodium species. This finding is in line with the malaria parasite distribution in Ethiopia [14]. It is also similar to those of studies conducted in Benna Tsemay district [15] and Nigeria [16]. Conversely, other studies conducted in East Shewa zone indicated that *Plasmodium vivax* was the dominant plasmodium species [9]. This might be so because *Plasmodium falciparum* is more widely distributed in most parts of Ethiopia. The possible discrepancy might be due to the fact that climate variation among study areas have effect on life cycle of parasite, parasite adaptation among *Plasmodium vivax* and falciparum.

This study also revealed that the odds of asymptomatic malaria parasitemia among male adults were more than in females. This result is also supported by previous studies conducted elsewhere [17, 18]. This might be due to the fact that in our setting, males are more exposed to outdoor activities than females and face increased risk of mosquito bit.

The odds of malaria infection among respondents in the age group of 15–19 years were higher compared with over 20 years (AOR = 4.5; 95%CI: 2.1–9.7). The finding is consistent with that of a study conducted in East Shewa which stated that being in the age group of 15–24 was more risks for malaria infection [9]. This might be due to the fact that participants in the 15 to 19 years groups are likely to have lower malaria sub-immunity than higher age groups. Moreover, higher exposure to outdoor activity of older adults before bed time might expose to malaria infections.

The utilization of ITN is a powerful vector control tool for reducing malaria transmission [13]. This study revealed that those who always slept under mosquito nets decreased the risk of malaria positivity compared to those who did not use ITN during bed time. This result is also supported by that of a study conducted in Hadiya zone which stated that those who did not use bed nets were 4.67 times more likely to be infected [3]. It is also supported by different studies conducted in different areas [4, 8, 16, 17, 19]. Environmental management for vector control is among the key components of the national malaria prevention and control strategy [13]. The study showed that individuals who had stagnant water around the house had a significantly increased risk of having malaria than those who had no such water. The result is also supported by that of a study conducted in south Ethiopia [4].

In this study, the odds of malaria infection among adults who had history of travel were higher than who did not travel away from their residence. This finding is similar with that of a study conducted in Jimma town, southwest Ethiopia [7]. This might be so because those who travel are less likely to use ITN and other protective techniques.

Though the study did its best to determine the prevalence of asymptomatic malaria parasitemia in the study setting, it is not free from some limitations. The cross-sectional nature of the study did not enable it to show temporal relationships. In addition, since the verification of malaria parasitemia was done using rapid diagnostic tests, it might have detected false results that did not indicate the actual status of the respondents. Furthermore, variables that were not considered in this study, such as occupation, marital status and residence were the other limitations of the study. Despite the aforementioned limitations, the study was able to assess malaria prevalence and associated factors in the community. This enables to show the relative precise figure of the problem and associated factors in the community, and thus, the result can be adequately generalized for similar population.

## Conclusion

Malaria, especially the predominant *Plasmodium falciparum*, is an important public health problem among the adult inhabitants of the study area. Males and those in the age group of 15–19 years are highly vulnerable groups for malaria positivity. ITN utilization is a protective factor for malaria infections, and factors like travel history and availability of stagnant water around dwelling are the risk factors. Therefore, during the implementation of malaria prevention and control activities, attention should be given to males who travel and work away from home and to youngsters in the age range of 15–19 years to use the most possible malaria prevention mechanisms. The District Health Office and health extension workers should be directed to work to increase ITN distribution in the community. The District Health Office should also focus on reducing or eradicating malaria breading sites through community participation. Travelers should use ITN, repellents, and protective clothes at places of arrival.

## Abbreviations

AOR: Adjusted odds ratio
CI: Confidence interval
COR: Crude odds ratio
IQR: Inter quartile range
IRS: Indoor residual spray
ITN: Insecticidal treated nets
RDT: Rapid diagnostic test
SPSS: Statistical package for social science
